# The Collaborative Metadata Repository (CoMetaR) Web App: Quantitative and Qualitative Usability Evaluation

**DOI:** 10.2196/30308

**Published:** 2021-11-29

**Authors:** Mark R Stöhr, Andreas Günther, Raphael W Majeed

**Affiliations:** 1 Justus-Liebig-University Giessen Universities of Giessen and Marburg Lung Center (UGMLC) German Center for Lung Research (DZL) Gießen Germany

**Keywords:** usability, metadata, data visualization, semantic web, data management, data warehousing, communication barriers, quality improvement, biological ontologies, data curation

## Abstract

**Background:**

In the field of medicine and medical informatics, the importance of comprehensive metadata has long been recognized, and the composition of metadata has become its own field of profession and research. To ensure sustainable and meaningful metadata are maintained, standards and guidelines such as the FAIR (Findability, Accessibility, Interoperability, Reusability) principles have been published. The compilation and maintenance of metadata is performed by field experts supported by metadata management apps. The usability of these apps, for example, in terms of ease of use, efficiency, and error tolerance, crucially determines their benefit to those interested in the data.

**Objective:**

This study aims to provide a metadata management app with high usability that assists scientists in compiling and using rich metadata. We aim to evaluate our recently developed interactive web app for our collaborative metadata repository (CoMetaR). This study reflects how real users perceive the app by assessing usability scores and explicit usability issues.

**Methods:**

We evaluated the CoMetaR web app by measuring the usability of 3 modules: *core module*, *provenance module*, and *data integration module*. We defined 10 tasks in which users must acquire information specific to their user role. The participants were asked to complete the tasks in a live web meeting. We used the System Usability Scale questionnaire to measure the usability of the app. For qualitative analysis, we applied a modified think aloud method with the following thematic analysis and categorization into the ISO 9241-110 usability categories.

**Results:**

A total of 12 individuals participated in the study. We found that over 97% (85/88) of all the tasks were completed successfully. We measured usability scores of 81, 81, and 72 for the 3 evaluated modules. The qualitative analysis resulted in 24 issues with the app.

**Conclusions:**

A usability score of 81 implies very good usability for the 2 modules, whereas a usability score of 72 still indicates acceptable usability for the third module. We identified 24 issues that serve as starting points for further development. Our method proved to be effective and efficient in terms of effort and outcome. It can be adapted to evaluate apps within the medical informatics field and potentially beyond.

## Introduction

### The Importance of Metadata

Raw data are useless without metadata that characterizes and contextualizes its content. A number is meaningless without the information on which parameter it describes (eg, blood pressure) and a finding is of no use without its context (eg, sepsis as a comorbidity vs sepsis as cause of death). Metadata itself always needs context (eg, the concept it describes). In many cases, metadata are merely implied by column headers of tabular databases and the implicit knowledge of the few people working with the database. Many information scientists have researched the field of metadata, for example, Wilkinson et al [1], who published the FAIR (Findability, Accessibility, Interoperability, Reusability) principles, which is a guideline for well-designed metadata. Whenever data are reused (for analysis, validity checks, etc), the corresponding metadata must be attached to the actual data. Thus, explicitly formulated, rich, and comprehensive metadata are indispensable for any sustainable research project [2]. At present, most data processing is done automatically by computers, which necessitates all metadata to be available in machine-readable form [3]. In addition to data processing, metadata are used to describe data sets to a broader audience, such as the national or international research community. BioPortal [4], for example, is a comprehensive repository of biomedical ontologies interconnecting researchers globally. In addition, there are approaches for recording the variety of existing data and metadata repositories in public registers [5,6].

### Metadata in the Field of Data Integration

#### Overview

Particularly in the context of data integration within large research networks, comprehensive metadata are essential. “Data integration is the problem of combining data residing at different sources, and providing the user with a unified view of these data” [7]. Although the process of exporting, transforming, and loading data is a huge task, this *unified view* is an achievement by itself. In medical informatics, the purpose of data integration is to promote translational research and to have access to a larger data pool for retrospective data analysis and prospective patient recruitment. The amount of integrated data and the way they are presented to users determine their acceptance and accessibility. If too few concepts are covered by a repository or if too few instances of data are integrated, researchers have no sufficient basis for analysis. If metadata are not presented accessibly, users will presume app shortcomings rather than investing in exploration time. This applies especially to entry-level users and, in most cases, yields in rejection of the software.

#### Data Integration: Main Components and Roles

Software-driven data integration involves multiple technical components: various *heterogeneous source databases* are harmonized and integrated into a *collective data repository*. All affected parameters, more precisely the canonical concepts behind these parameters, are annotated in a separate *metadata repository*. Both repositories are linked through identifiers [8-10]. *Configuration files* define the harmonization process of different source database schemata into a target schema. These configuration files vary in format and syntax, but all of them are written in a formal computer-readable language [11-14].

From the user perspective, these components are managed and elaborated by the following roles: *data providers* know the meaning of their data and its acquisition processes. In medical informatics, this knowledge is essential for data harmonization, because labels such as column names or form labels are not always sufficiently specific. According to Nadkarni and Marenco [15], “[...] column names may be quasi-gibberish, heavily abbreviated, and their names may follow arbitrary conventions that are idiosyncratic to the system designer or organization.” Rahm and Bernstein [16] showed that even automatic schema matching can only provide mapping candidates. The formulation of mapping rules is performed by the *local and central data managers* (responsible for the source databases and collective database repository, respectively) as they have the required technical background to maintain the formally written configuration files. *Data coordinators* elaborate the metadata repository content, incorporating multiple studies and registers with varying scopes and the focus of research. This process includes rating for relevance, harmonization, annotation, curation, and clustering. The clustering and hierarchical organization of metadata have a direct impact on the presentation of user interfaces. It determines how intuitively information can be found and used.

#### Information Access Barriers

To provide a data warehouse with comprehensive and accurate data, different roles need access to different classes of information residing in the described data integration system. We identified 3 cases in which access barriers prevent users from contributing their expertise [17,18]:

1. All users need access to the listing of all data elements represented in the data warehouse. These annotations and context information can be derived from the metadata repository and must be visualized.
2. Data managers and, in particular, data providers need full access to the mapping rules for data harmonization. They are only available in the formal language, which requires the respective information technology background. Data providers usually do not have that knowledge.
3. Data coordinators need access to the provenance information of the metadata to be able to curate it. “Especially in collaborative metadata development, a comprehensive annotation about ‘who contributed what, when and why’ is essential” [17].

In most cases, barrier (1) is resolved through metadata browsers [4,19,20]. For metadata repositories in the context of data integration, barriers (2) and (3) often form a huge gap between users and the required information.

### The Implementation of Collaborative Metadata Repository

The German Center for Lung Research (German: Deutsches Zentrum für Lungenforschung [DZL]) implemented the collaborative metadata repository (CoMetaR), applying principles of collaborative metadata development and FAIR metadata warehousing [1,17,18,21]. It is based on open and commonly used standards. The DZL metadata constitutes a highly specified thesaurus specifically developed for lung research, and till July 2021, it contains 3.474 distinct concepts. CoMetaR supports storing a single thesaurus in the Resource Description Framework (RDF) format based on the Simple Knowledge Organization System (SKOS) and Dublin Core (DC) knowledge organization systems [22-24]. The ISO/IEC 21526 [25] standard explicitly “mandates the use of SKOS to provide user-interface surfaced content classification.” Versioning occurs via Git, which also provides information about the changes among different versions [26,27]. The latest thesaurus version is loaded in a triple store and accessible through the SPARQL Protocol and RDF Query Language (SPARQL) interface [28]. This interface can be used to extract metadata information and, as in our case, to set up tree-like metadata in a data warehouse [29]. The extracted metadata information can also be used to generate a visual metadata representation similar to our user front end, the CoMetaR web app. This front end was developed to dissolve access barriers for all user roles and thereby support them in contributing to their expertise. However, it has yet to be proven scientifically that the CoMetaR web app meets the requirements for metadata management and data integration support.

This study evaluates the usability of 3 modules built for common tasks in the field of data integration and metadata maintenance.

## Methods

### Study Design

#### Overview

The usability evaluation performed was a combination of (1) the think aloud method and (2) usability questionnaires. By combining both methods, we wanted to measure both observable and perceived usability. The execution consisted of two phases: (1) a screen sharing–supported training specific to the respective user’s roles and (2) solving of the given tasks by the participant with subsequent retrospection, including the completion of a usability questionnaire. All evaluations were performed by the same experimenter.

#### The Think Aloud Method

This method is commonly applied to the usability evaluations of web interfaces [30,31]. The idea behind the think aloud method is that participants verbalize their thoughts while performing given tasks. Their expressions were recorded and later transcribed and analyzed according to an interpretation model.

We decided not to record the participants but to make notes on their expressions as well as their app use behavior. These notes focused on usability, functional, and methodological issues. The advantage of this approach is a more comfortable setting for the user on the one hand and less effort for the experimenter on the other hand. The downside is the potential information loss because the experimenter already filters information.

As our interpretation model, we used the 7 categories described in ISO 9241-110 [32]: suitability for the task, conformity with user expectations, suitability for learning, suitability for individualization, self-descriptiveness, controllability, and error tolerance.

#### System Usability Scale

We used the System Usability Scale invented by Brooke in 1996 as a measurement tool for the usability of the app. This scale was introduced as a *quick and dirty* but a meaningful measurement tool for user experience [33,34]. It consists of 10 questions answered on a scale from 0 to 4. All questions are available in multiple languages, including German, which we used for our evaluation.

#### Materials

##### CoMetaR Modules

**Figure 1:**
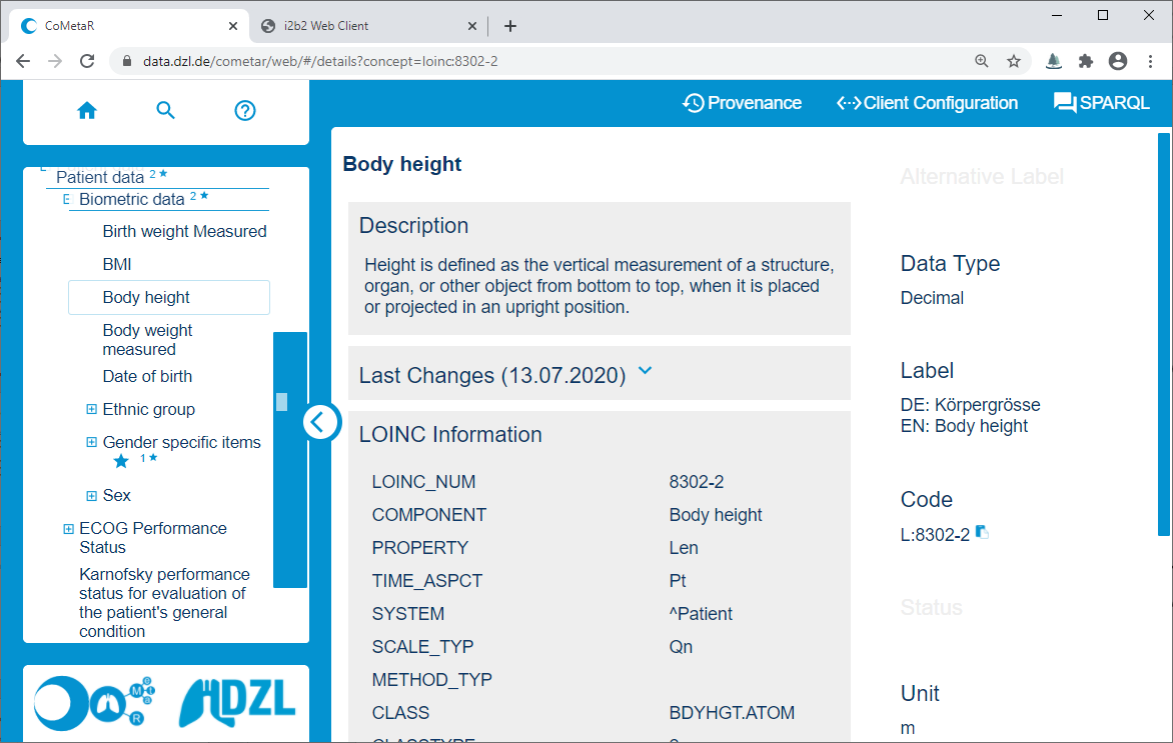
Screenshot of the collaborative metadata repository (CoMetaR) web app core module. Left side: concept tree. Right side: module content (concept details). Top-right corner: module navigation. Top-left corner: home button, search panel, and help panel.

The CoMetaR web app is divided into a concept tree navigation area and a module area. Modules can be selected in the module menu in the top-right corner, as shown in Figure 1. In the following paragraphs, we will briefly describe the functionality of the 3 evaluated modules: the *core module*, *provenance module*, and *data integration module*. In the *Introduction* section, we described 3 user roles involved in the data integration process: data managers, data providers, and data coordinators. A user may perform more than one role. Each role makes use of the core module, whereas the data integration module and provenance module are more role-specific (see the Tasks section).

The core module functionality of the CoMetaR web app (Figure 1) involves browsing through all metadata concepts and showing the corresponding detailed information. Users can navigate the concept tree by expanding the nodes and retrieving details by clicking them. They can also use the search function to check if and where a concept is located in the thesaurus. Concept details are shown in the module area. They include core information like labels, alternative labels, data type, code, status (*on draft* yes or no), and unit. In addition, we present the author, description, and concept specifications. A dedicated panel shows the history of all changes that have been made to the selected concept. A button allows the export of the concept and all of its subconcepts with basic information in the CSV format.

**Figure 2:**
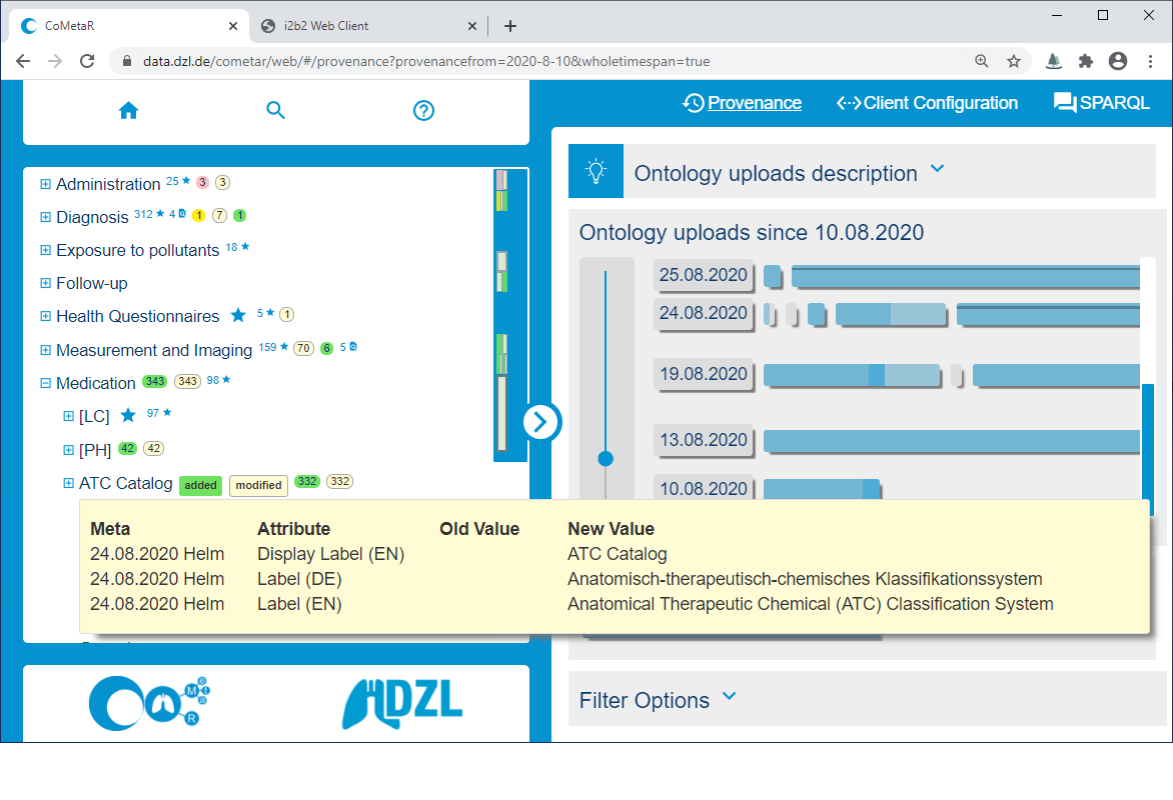
Screenshot of the collaborative metadata repository (CoMetaR) web app provenance module. Left side: concept tree with colorized annotations for added, moved, or removed and modified items. Light yellow box: information box for the item ATC Catalog on mouse-over. Right side: module content (upload history visualization). ATC: Anatomical Therapeutic Chemical.

As our metadata are growing and developing over time with many participants involved, we decided to provide the provenance module, which enables users to track all changes. These changes may be the additions, moves, or removal of concepts in the concept tree, but also modifications of their annotations. When selecting the provenance module (Figure 2), the affected concept tree elements receive icons that symbolize their changes for a given timespan. The default timespan is 1 month from the current date and can be adjusted in the module. The module itself shows all dates concerned with metadata changes in vertical order. Horizontal bars attached to such a date represent single uploads, and their width indicates the amount of change. Clicking a date or a single upload bar loads the respective changes and shows them in the concept tree underneath the corresponding concepts.

**Figure 3:**
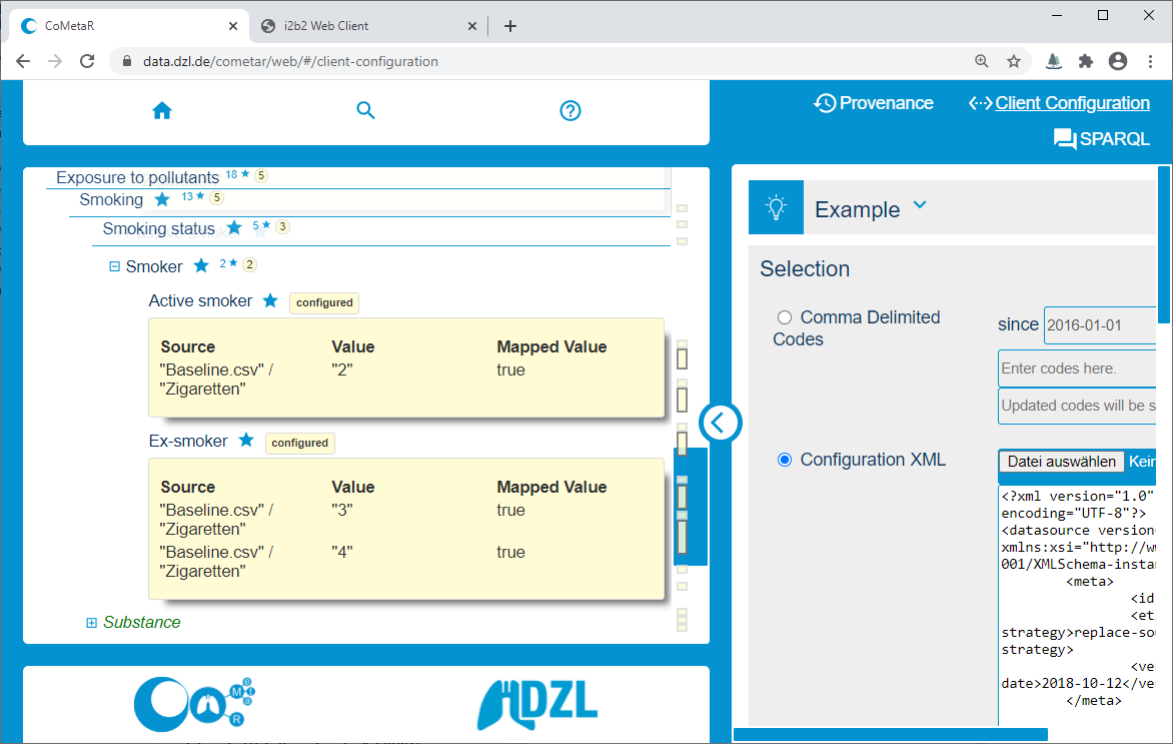
Screenshot of the collaborative metadata repository (CoMetaR) web app data integration module. Left side: concept tree. Light yellow boxes: corresponding mapping rules. Right side: module content (configuration file upload).

Our data integration process is supported by the data integration module. The integration process for a single data source is divided into 4 parts. (1) The export of data from the source system, (2) the preparation of data for the integration software, (3) configuration of the integration software, and (4) its execution. As the configuration file is written in formal language to be interpreted by software, it is not accessible for humans who lack the required technical background. To verify the configurations, the respective data providers must be able to access the formulated rules. For this task, they can upload the configuration file to the data integration module (Figure 3). All rules are then shown below the corresponding concept in the concept tree. In addition, we print notifications in the module area if any rule refers to a concept that does not exist (anymore) or that has been reintroduced. In such a case, the correct reference can be determined automatically, depending on the metadata’s formal documentation. Subsequently, an updated version of the configuration file is offered for download. Note that this process does not invoke any kind of data upload; it is solely used to verify the configuration itself.

##### Tasks

CoMetaR was designed to support data integration tasks. In the German Center for Lung Research, we have been practicing data integration since 2016 and identified information that is of high interest for data integration experts. For example, to match and map elements of the source data to the integrated data, the person formulating the rules needs to know which elements are part of the integrated metadata, what are their exact characteristics (method of measurement, scale, classification, etc), and how they are uniquely identified. If these characteristics change, the mapping rules must be adjusted. For various processes, people often want the metadata to be available in Microsoft Excel format, yielding the need for respective export capabilities. For these and further scenarios, we defined 10 tasks that verified CoMetaR’s suitability in the field of lung research. The following tasks were composed by 2 experts, who have been internationally active in the field of data integration for >5 years. The composition process included brainstorming, discussion, and finally consensus. To assign modules to each participant, we considered their user roles as well as their everyday tasks. All users must solve core module tasks, all data coordinators must solve provenance module tasks, and all data managers who upload data must solve the data integration module tasks.

The first 4 tasks aim at the use of the core module. They test the ability to search for and find specific thesaurus elements and their annotations as well as the capability to export data:

1. Indicate which of the parameters *Never smoker* and *Opportunity smoker* are part of the DZL metadata.

2. Indicate code, datatype, and unit of the spirometry parameter Forced Expiratory Volume in 1 Second (*FEV1*) according to the metadata.

3. Regarding the last change of the concept *Comorbidities*, indicate its date and the modifications applied.

4. Describe in detail which individual steps you would take to print the subtree of *Biometric Data* in tabular form.

The following 2 tasks aim at the use of the provenance module. They test the ability to track changes within the thesaurus:

5. Indicate which concepts have been added, moved, or removed in the last month.

6. Pick one concept for which annotations have been changed in the last upload. Indicate who performed this change on which date.

The last 4 tasks aim at the use of the data integration module. They test the ability to verify individual upload client configurations:

7. Examine the configuration for falsely mapped concepts.

8. Examine the configuration for properly mapped concepts.

9. Examine the metadata for concepts that are not mapped in the configuration but you could provide.

10. Update your local configuration to meet changed concept references. Describe your approach.

Tasks 7, 8, and 9 must be seen as one task with 3 subtasks. The participants were asked to use their own configuration files designed for uploading the data they administered. Some configuration files comprise hundreds of mapping rules. Depending on the size and coverage of certain data sources, task fulfillment takes a considerable amount of time. During the live evaluation, the participants were asked to work on each of these 3 tasks exemplarily to be able to fill out the System Usability Scale questionnaire. They completed the tasks asynchronously and reported their results when they finished.

##### Configuration Files

For 3 of the 4 data integration module tasks, we asked the participants to use their own configuration file for analysis. These comprise rules to define how local concepts are mapped to concepts in the central data warehouse. The file format is XML. The configuration files are used by a data transformation and upload client software. Configuration files do not contain any instance data. By using real configuration files instead of an artificial example, we were able to test our app in a realistic scenario and identify faulty mappings. In addition, this setup allowed participants to work with familiar information.

##### Experimenter Notes

The experimenter completed a notes sheet alongside following the evaluation procedure. It was structured to contain one row per participant and the following columns: *Experience level*, *English level*, *age*, *profession*, *roles* (see the *Introduction* section), *evaluation date*, *training start timestamp*, *training finished timestamp*, *notes for training*. Each of the 3 modules contains the following columns: *module tasks* (stating whether tasks were solved successfully), *module finished timestamp*, *notes for module*, *timestamp module questionnaire filled*.

##### System Usability Scale Questionnaires

The questionnaires handed to the participants contained 10 usability questions defined in the System Usability Scale. They were put into a Microsoft Excel sheet with one row for each question and columns for values of 0 to 4. The final score for the 10 questions was calculated within the sheet. The participants were handed one sheet per evaluated module.

##### Quantitative Analysis Sheet

A spreadsheet was used to collect the scores per participant and module to calculate the quantitative analysis parameters, that is, *range from*, *range to*, *mean score*, and *SD*. These 4 parameters were additionally calculated with respect to the participant’s experience level, using the following formula:

Score weighted by experience = score − 4 × (experience level−1)

Given an experience level from 1 to 5, the score weighted by experience differs by up to 16 points, which corresponds to previous findings [35]. In addition to scores from the questionnaires, the corresponding experience levels, and the calculated values, no participant-related information was put into the sheet.

#### Setting

To evaluate our web app, we decided to interact with the participants remotely (participants were not invited to a local test laboratory) and synchronously (the evaluator and participant executed the test session in real time). We made one exception for a very time-consuming task type, which certain participants completed asynchronously. This method appeared to be the most efficient in terms of preparation effort, travel time, and risk of SARS-CoV-2 infection. Its suitability was shown in a comprehensive study: Bastien [36] summarized multiple studies stating that remote evaluations yield comparable results with a local laboratory evaluation. Although he found that automatic recording of every user interaction with the app can provide more insights about the app’s usability, the setup is very time-consuming and would only be rational for larger participant numbers. The participants were approached in April and May of 2020. Data collection took place in May and June of 2020. Data analysis was conducted in July of 2020.

As a communication platform, we used the GoToMeeting web conference software by LogMeIn [37]. It allows participants to dial in via phone or software app. The latter also offers screen sharing capabilities, which all but one participant with technical issues were able to use.

#### Sampling

The target audience of CoMetaR is experts who contribute to the task of data integration as data providers, data managers, or data coordinators. Our implementation of CoMetaR is dedicated to lung research. Therefore, in this evaluation, we included members of the German Center for Lung Research and collaborating organizations. The included participants should cover a wide range of roles and responsibilities. These characteristics determine the module that they can work on effectively. For example, data managers who load data into a data warehouse have a data integration configuration file and can use the data integration module. The core module is relevant to all the user roles. In contrast, the provenance module is mostly relevant for data coordinators and data managers, whereas the data integration module is mostly relevant for data managers and data providers. In addition to their user role, profession, age, and English level, we also asked for the participants’ experience with the app. English and experience levels were measured on a scale of 1 to 5.

Bastien [36] cited studies showing that most usability problems can be found in 5-15 participants. As Virzi [38] showed, only 4 to 5 participants were needed to identify about 80% of all usability issues, and this number is enough to reveal the most severe issues. Therefore, we planned to recruit at least 5 participants for each aspect of the web app. In total, we approached 13 potential participants, of whom 12 agreed to participate.

#### Ethical Considerations

All methods were performed in accordance with the relevant guidelines and regulations. This study was granted an exemption from requiring ethics approval by the ethics committee of the Faculty of Medicine at the Justus-Liebig-University in Giessen, Germany. Informed consent for participation in the study was obtained from all the participants.

All patient-related data were recorded anonymized. It covers age, profession, role, evaluated modules, English level, and experience with the app. The data were further coarsened using age classes of 10 years to prevent participant reidentification.

### Procedure and Data Collection

#### Overview

Before any evaluation, we performed a screen sharing–supported training specific to the respective user’s roles, regardless of previous experiences with the app. The goal of this training was to provide participants with equal basic knowledge about the web app’s structure and functionality. We asked for the participants’ previous experiences with the system, which may influence the evaluation outcome [35]. After the training, the participants shared their screens and completed the tasks given by the evaluator. After using the module, the participants filled out the System Usability Scale questionnaire. This also gave them the chance for retrospection and a short dialogue with the experimenter, potentially revealing more usability issues.

#### Instructions

After giving each participant introductory training regarding the app’s functionalities, they had the option to ask questions and clarify misunderstandings. Following, for each tested module, they were asked to fulfill each task one by one. The tasks were communicated via speech. The experimenter asked the participants to verbalize their thoughts during the evaluation and reminded them whenever they forgot. After the participant solved the tasks for a module, the experimenter asked them to fill out the usability questionnaire we sent them previously via email. Furthermore, they were invited to participate in a retrospective dialogue, again noting the findings.

#### Role of the Experimenter

The experimenter played a passive role. During the evaluation, he was not supposed to speak besides reminding the participant to verbalize their thoughts. In cases where the participants were stuck, the experimenter gave hints to lead to the information that had to be received from the app. Meanwhile, the experimenter completed the structured notes sheet documenting the participants’ verbalized thoughts, spontaneous reactions, and their app use behavior, focusing on the previously mentioned usability categories [32].

#### Recording and Transcription

The traditional think aloud method requires recording the entire evaluation session and the following transcription. As mentioned in the study design, we did not record sessions because transcription occurred during the session.

### Analysis

#### Quantitative Analysis

For quantitative analysis, we calculated aggregated scores (*range from*, *range to*, *mean score*, and *SD*) based on the System Usability Scale questionnaires. We additionally calculated the same aggregations factoring in the experience level. This adjustment is motivated by previous findings, which show that usability scores vary up to 16 points based on the participant’s experience level [35]. For example, a user with no experience (level 1) has the same base and adjusted score, whereas a user with a score of 70 and experience level 4 (of 5) has an adjusted score of 58. By calculating these moderated scores, we hope to obtain better insights into the app’s usability, especially regarding entry barriers. All calculations were performed using Excel (see *Materials* section). We omitted subgroup analysis by English level, age, and profession as our sample size was too small.

#### Qualitative Analysis

We conducted a thematic analysis of the information gathered during the evaluations to identify usability issue patterns and to present a descriptive account of users’ experiences. After familiarization with all notes, we went through all notes again and generated usability issue statements. We followed a latent approach, which means that we interpreted the data to create statements that were more meaningful. For example, task 2 asked the participants to indicate the properties of the spirometry parameter *FEV1*. In one case, a participant used the search function and entered *Spiro FEV1*, which led to no results (a note in the experimenter’s structured notes file). Our conclusion is not that our app is unable to find a specific pattern but that users expect a more powerful search functionality, as is known from bigger internet companies (theme). After generating usability issue themes, we combined similar statements and reviewed them by checking if all notes were still well-represented by these statements. These were then assigned to 1 of the 7 usability categories described in ISO 9241-110 [32]: suitability for the task, conformity with user expectations, suitability for learning, suitability for individualization, self-descriptiveness, controllability, and error tolerance. The categorization was performed by the same person who underwent the evaluation sessions with all participants. Afterward, these groupings were discussed internally with another expert and potentially adjusted.

#### Software

For documentation and analysis, we used only Microsoft Excel and Microsoft Word.

#### Quality Assurance

The System Usability Scale questionnaire consists of 10 questions, 5 of which stated a positive usability and 5 of them stated negative usability. As some questions include negations, we assumed a possible misinterpretation. Therefore, we immediately checked each questionnaire for outliers and inquired when we identified potential misinterpretations. When inquiring, we again pointed out that we do not insist on better scores but on valid answers.

We wanted to ensure correct and comprehensive categorization, as well as unambiguous wording for qualitative analysis. A second person who was familiar with the study design and aspects of usability checked all categorizations. The resulting tables are the results of in-depth dialogues.

## Results

### Participants

All participants in this evaluation currently work for or in collaboration with the German Center for Lung Research. Their operation areas and responsibilities vary, but all contribute to the data integration task. Table 1 shows the details of all the 12 participants. They vary in age (28-63 years), experience with the system (1-4 on a scale of 1-5), English level (2-5 on a scale of 1-5), and profession (medical documentalists, medical informatics specialists, graduated biologists, bioinformatics specialists, study coordinators, and data managers).

**Table 1:** Characteristics of the 12 participants including age, experience level, English level, profession, user roles, and tested modules.

Characteristics	Participants											
	A	B	C	D	E	F	G	H	I	J	K	L
Age (years)	30-40	30-40	30-40	40-50	50-60	60-70	30-40	50-60	30-40	50-60	60-70	20-30
Experience level (1-5)	3	3	4	2	4	3	3	3	3	1	2	4
English level (1-5)	3	3	4	3	4	4	4	5	3	3	2	4
Profession	MD^a^	DM^b^	MI^c^	SC^d^	MD	GB^e^	MI	DM	DM	MD	MD	BI^f^
Has role data manager	✓^g^	✓	✓	✓				✓	✓	✓	✓	✓
Has role data provider	✓	✓	✓			✓						
Has role data coordinator	✓	✓	✓		✓	✓	✓					
Tested core module	✓	✓	✓	✓	✓	✓	✓	✓	✓	✓	✓	✓
Tested provenance module	✓	✓	✓		✓	✓	✓	✓	✓	✓		✓
Tested data integration module	✓	✓	✓	✓					✓			

^a^MD: medical documentalist.

^b^DM: data manager.

^c^MI: medical informatics specialist.

^d^SC: study coordinator.

^e^GB: graduate biologist.

^f^BI: bioinformatics specialist.

^g^Characteristic present.

### Quantitative Analysis

#### Time Expenditure

The training took between 10 and 30 minutes, depending on how many modules were presented and how many questions the participants had. After training, for task completion, the core module took between 8 and 26 (average 14, SD 6) minutes. The provenance module took between 3 and 20 (average 9, SD 5) minutes. The configuration module took between 21 and 51 (average 37, SD 12) minutes. Regarding the latter, we did not include the time spent asynchronously to complete the tasks.

#### Usability

Each participant solved the tasks of one or more CoMetaR modules (core module n=12, provenance module n=10, data integration module n=5). Subsequently, they completed one System Usability Scale questionnaire separately for each module. According to Bangor et al [39], a mean score of 50.9 or higher can be seen as *OK*, a mean score of 71.4 or higher is *Good*, and a mean score of 85.5 or higher is *Excellent*; a mean score of 70 or higher indicates that the interface is acceptable. The System Usability Scale score results are presented in Table 2. For *weighted by experience*, we subtracted up to 16 points based on the user’s own perceived experience.

**Table 2:** Aggregated System Usability Scale scores.

Module and score type		Values, mean (SD; range)
**Core module**		
	Usability score	81.5 (9.1; 60.0-92.5)
	Weighted by experience	73.8 (7.8; 60.0-84.5)
**Provenance module**		
	Usability score	72.3 (16.0; 37.5-90.0)
	Weighted by experience	63.9 (15.20; 37.5-79.5)
**Data integration module**		
	Usability score	81.0 (9.9; 65.0-92.5)
	Weighted by experience	73.0 (9.9; 57.0-84.5)

#### Functional Suitability

All the participants successfully solved all given tasks. In total, 12 participants solved 48 core module tasks, 10 participants solved 20 provenance module tasks, and 5 participants solved 20 data integration module tasks. In the case of task 2, 2 participants did not find the correct tree node and needed a hint. During the provenance module tasks, 1 participant lost track because he loaded too much information from multiple modules into the tree. He needed a hint to reset the app to solve task 5. In total, 97% (85/88) of all tasks were solved independently.

### Qualitative Analysis

Our thematic analysis led to 24 usability issue themes, which covered all functional inadequacies and complications identified during the experiment. We grouped these themes into the 7 categories described in ISO 9241-110 (Textboxes 1-5). As the app does not offer possibilities for individualization, the respective category *suitability for individualization* does not appear in this evaluation. None of the observed issues were assigned to the category *controllability*.

**Issues in the category *Suitability for the Task*.**

**Core module**

- Using the search function for *FEV1* shows more than 100 results because it is used as criterion for many diseases. Most of the results are located in the comorbidities-subtree.
- The help window does not help with task 2.

**Provenance module**

- The mouse-over tooltip of upload bars sometimes distracts and overlays other bars.
- Changing the selection of upload bars leads to changes in the concept tree. The system gives insufficient feedback that these changes were applied.

**Issues in the category *Conformity with User Expectations*.**

**Core module**

- The search function only searches for fixed substrings and does not behave comparably to a mighty World Wide Web search engine. This might lead to incorrect conclusions whether a concept is part of the metadata.
- The users expected the fixed headings for the currently displayed subtree to be interactive.

**Provenance module**

- The provenance module disappears when clicking a tree element and the element’s core information are shown instead.

**Issues in the category *Suitability for Learning*.**

**Core module**

- An element’s change history is part of the core module and not the provenance module.
- Structural information for elements (added, moved, or removed) are not explicitly displayed in the element’s history (last changes).
- The number of search matches is not the number of matched concepts but of all matched attributes.
- Some annotations like *added* have rectangular representation in the minimap or outline and round-cornered representation in the tree.

**Provenance module**

- The structural annotations (added, moved, or removed) refer to the selected provenance timespan and not only to the selected uploads.
- It is not intuitive that a moved element’s old and new concept tree position are both selected when clicking one of them.

**Issues in the category *Self-Descriptiveness*.**

**Core module**

- Many people search the code for *Forced Expiratory Volume in 1 Second* in the *Logical Observation Identifiers Names and Codes (LOINC)*–description instead of the concept’s core information.
- For some users, it is not intuitively clear that details for a tree node are shown when clicking them.
- Symbols in the tree are not explained through a legend, but only mouse-over tooltips.
- The minimap or outline next to the scrollbar is not intuitive for users that are not familiar with such.
- The scroll bar is differently styled than a standard scroll bar and might not instantly be recognized as such.

**Provenance module**

- For some users, it is not noticeable whether an upload was selected.
- The function of the *load all changes* button is not clear.
- The temporal order (left to right or right to left) of multiple uploads on the same day is not clear.

**Data integration module**

- For elements with more than one configuration rule, it is not intuitive that the rules are applied from top to bottom order.

**Issues in the category *Error Tolerance*.**

**Core module, provenance module, and data integration module**

- Activating multiple modules and searches leads to an overload of information in the concept tree.
- Loading too many information into the tree and expanding many of affected tree elements leads to high central processing unit (CPU) use.

## Discussion

### Principal Findings

In total, 12 participants took part in the evaluation of up to 3 modules of the CoMetaR web app, and each participant completed up to 10 tasks; 97% (85/88) of all tasks were solved independently and successfully. The core module and data integration module both obtained a mean usability score of 81, which proves good and nearly excellent usability. For inexperienced users, we estimated a mean usability score of 73, which proves good and acceptable usability. The provenance module has a mean usability score of approximately 72, which implies good and acceptable usability. For inexperienced provenance module users, we estimated a mean usability score of 63, which indicates unacceptable usability. We identified 24 issues with the app, which we grouped into 5 usability categories based on ISO 9241-110. From our point of view, of particular note are (1) information displayed in the concept tree can be overwhelming, especially if information from multiple modules is shown at once. (2) For many users, the provenance module and its functionalities are not accessible. The number of options, such as filtering by timespan or upload package, demand an extensive introduction and learning period. (3) The search functionality can output far more hits than expected because every literal information about concepts is considered. Some sort of categorization or filtering may be useful.

### Strengths and Limitations

The strength of our study design is the relationship between effort and outcome. Although we omitted the step of recording audio and video of each session, we found a considerable compilation of usability issues and clear quantitative categorization of our tested modules owing to the System Usability Scale questionnaire. All testing sessions were performed by a single experimenter. For thematic analysis, an additional scientist was consulted.

Retrospectively, we identified 4 problems regarding the evaluation methodology. The web conference software used in this evaluation was always visible and, in some cases, overlapped crucial information in the browser window. Second, one person tried to participate via an Apple product and was not able to establish screen sharing because of missing technical literacy. The third problem concerns communicational logistics, specifically around task instructions being communicated verbally by the evaluator. Some participants missed important aspects of the tasks because they were inattentive or started solving the tasks before the instruction was finished. Finally, some tasks were not formulated in sufficient detail. For example, for task 5, a participant thought it would be sufficient to read the respective upload description, but we expected them to list all changes explicitly in detail.

We did not record audio and video, for which reason we probably missed single verbalizations and observations. Thus, we cannot claim that our list of usability issues is complete at 100%, which arguably is never the case. In addition, the experimenter already filtered information during the test sessions, which might have biased the qualitative analysis outcome. We still assume that we found most usability issues, especially the most severe ones, because the experimenter was able to follow every action throughout all sessions without difficulty.

As all tasks were performed in our production environment, the upload history and thus the collection of added, moved, or removed or modified concepts varied. This may have led to differing results among the participants. We assumed that these differences were negligible in the usability evaluation.

### Comparison With Previous Work

In 2009, considering 317 web apps, Bangor et al [39] found that web apps have a mean usability score of 68.2, which confirms the above-average usability of our app. Owing to increased awareness regarding usability, these values might have changed, but we did not find a more recent usability score meta-analysis. To the best of our knowledge, our approach to calculate another score for inexperienced users has not been done before. It allows the assessment of usability scores for inexperienced or new users even though some participants already have experience with the app.

Regarding the think aloud method, it is usual to record and transcribe all user sessions. Other studies show that this consumes a considerable amount of time and labor, which is often done by multiple scientists. In addition, we did not count code quantities within a transcript, as this is often done in a thematic analysis. We adopted the highest-level themes from an ISO standard instead of creating them ourselves.

### Implications and Future Work

After evaluating our app, we are able to improve it by addressing all found usability issues. This will, in the first place, improve research in the field of lung research because lung research–specific metadata availability and accessibility will be improved. This app has already been considered by other German Centers for Health Research. We hope to be able to generally improve the field of health research.

Second, we applied a methodology that allows the usability evaluation of metadata management apps with a considerably low effort in time and labor. In an adapted form, this method can be applied to similar apps. Although the first 4 tasks of our evaluation are specific to the field of lung research concerning content, their content-agnostic intention is to check if basic information can be retrieved from the app. This includes the existence and findability of concepts (task 1), identification of a concept’s annotations (task 2), its development over time (task 3), and the export of information about a unit of concepts (task 4). The application programming interface for the data integration module is specific to our data integration configuration file format, but the tasks represent the crucial steps to be taken to verify such a configuration file. The next step for this project could be the application of this evaluation method to comparable apps to approve its reliability and to find common usability issues.

We also hope that the findings of our qualitative analysis raise other developers’ awareness of possible shortcomings in their own apps. For example, they might also plan to visually annotate concepts in the concept tree, in which case we highly recommend not displaying too much information at once.

A potential alternative or addition to the think aloud method with a thematic approach could be a heuristic evaluation performed by usability experts. The advantages and disadvantages of both methods were researched by Yen and Bakken [30].

We experienced issues with the web conference software, whose control panel sometimes overlapped crucial information on the user display. For further remotely and synchronously performed evaluations, we recommend ensuring that all relevant web app content is always visible, for example, by choosing different conference software.

We found that the assumed average usability score for inexperienced users was approximately 8 points lower than the original average score. This implies, on the one hand, that entry barriers exist within the app. On the other hand, these barriers can at least partly be overcome with experience. Measuring such a score might be of special interest for apps that provide a more efficient alternative to existing methods of information retrieval. Entry barriers may lead to rapid rejection of the entire software.

### Conclusions

Our goal was to find usability issues of the CoMetaR web app and to measure its usability as perceived by real users. We identified 24 issues, which will be starting points for app improvement. On average, the app was assessed as good and in parts nearly excellent in terms of usability. Our method proved effective and efficient in terms of effort and outcome. Future research should improve our app and evaluate similar solutions. We invite other researchers interested in evaluating biomedical metadata repositories to adapt our methodology. All source codes are publicly accessible under GitHub [40]. The production instance of the German Center for Lung Research metadata repository is publicly accessible [41].

## Abbreviations

CoMetaR: collaborative metadata repository
DC: Dublin Core
DZL: Deutsches Zentrum für Lungenforschung
FAIR: Findability, Accessibility, Interoperability, Reusability
FEV1: Forced Expiratory Volume in 1 Second
RDF: Resource Description Framework
SKOS: Simple Knowledge Organization System
SPARQL: SPARQL Protocol and Resource Description Framework Query Language

## References

[1] Wilkinson MD, Dumontier M, Aalbersberg IJ, Appleton G, Axton M, Baak A, Blomberg N, Boiten J, da Silva Santos LB, Bourne PE, Bouwman J, Brookes AJ, Clark T, Crosas M, Dillo I, Dumon O, Edmunds S, Evelo CT, Finkers R, Gonzalez-Beltran A, Gray AJ, Groth P, Goble C, Grethe JS, Heringa J, 't Hoen PA, Hooft R, Kuhn T, Kok R, Kok J, Lusher SJ, Martone ME, Mons A, Packer AL, Persson B, Rocca-Serra P, Roos M, van Schaik R, Sansone S, Schultes E, Sengstag T, Slater T, Strawn G, Swertz MA, Thompson M, van der Lei J, van Mulligen E, Velterop J, Waagmeester A, Wittenburg P, Wolstencroft K, Zhao J, Mons B (2016). The FAIR Guiding Principles for scientific data management and stewardship. Sci Data.

[2] Kush RD, Warzel D, Kush MA, Sherman A, Navarro EA, Fitzmartin R, Pétavy F, Galvez J, Becnel LB, Zhou FL, Harmon N, Jauregui B, Jackson T, Hudson L (2020). FAIR data sharing: the roles of common data elements and harmonization. J Biomed Inform.

[3] Hume S, Chow A, Evans J, Malfait F, Chason J, Wold JD, Kubick W, Becnel LB (2018). CDISC SHARE, a global, cloud-based resource of machine-readable CDISC standards for clinical and translational research. AMIA Jt Summits Transl Sci Proc.

[4] Noy NF, Shah NH, Whetzel PL, Dai B, Dorf M, Griffith N, Jonquet C, Rubin DL, Storey M, Chute CG, Musen MA (2009). BioPortal: ontologies and integrated data resources at the click of a mouse. Nucleic Acids Res.

[5] Sansone S, McQuilton P, Rocca-Serra P, Gonzalez-Beltran A, Izzo M, Lister AL, Thurston M, FAIRsharing Community (2019). FAIRsharing as a community approach to standards, repositories and policies. Nat Biotechnol.

[6] Pampel H, Vierkant P, Scholze F, Bertelmann R, Kindling M, Klump J, Goebelbecker H, Gundlach J, Schirmbacher P, Dierolf U (2013). Making research data repositories visible: the re3data.org Registry. PLoS One.

[7] Lenzerini M (2002). Data integration: a theoretical perspective. Proceedings of the twenty-first ACM SIGMOD-SIGACT-SIGART symposium on Principles of database systems.

[8] Zhang H, Guo Y, Li Q, George TJ, Shenkman E, Modave F, Bian J (2018). An ontology-guided semantic data integration framework to support integrative data analysis of cancer survival. BMC Med Inform Decis Mak.

[9] Stathias V, Koleti A, Vidović D, Cooper DJ, Jagodnik KM, Terryn R, Forlin M, Chung C, Torre D, Ayad N, Medvedovic M, Ma'ayan A, Pillai A, Schürer SC (2018). Sustainable data and metadata management at the BD2K-LINCS Data Coordination and Integration Center. Sci Data.

[10] Dugas M, Meidt A, Neuhaus P, Storck M, Varghese J (2016). ODMedit: uniform semantic annotation for data integration in medicine based on a public metadata repository. BMC Med Res Methodol.

[11] Ong TC, Kahn MG, Kwan BM, Yamashita T, Brandt E, Hosokawa P, Uhrich C, Schilling LM (2017). Dynamic-ETL: a hybrid approach for health data extraction, transformation and loading. BMC Med Inform Decis Mak.

[12] Pecoraro F, Luzi D, Ricci FL (2015). Designing ETL tools to feed a data warehouse based on electronic healthcare record infrastructure. Stud Health Technol Inform.

[13] Post AR, Krc T, Rathod H, Agravat S, Mansour M, Torian W, Saltz JH (2013). Semantic ETL into i2b2 with Eureka!. AMIA Jt Summits Transl Sci Proc.

[14] Post AR, Pai AK, Willard R, May BJ, West AC, Agravat S, Granite SJ, Winslow RL, Stephens DS (2016). Metadata-driven clinical data loading into i2b2 for Clinical and Translational Science Institutes. AMIA Jt Summits Transl Sci Proc.

[15] Nadkarni P, Marenco L (2014). Chapter 2 - data integration: an overview. Methods in Biomedical Informatics: A Pragmatic Approach.

[16] Rahm E, Bernstein P (2001). A survey of approaches to automatic schema matching. The VLDB J.

[17] Stöhr MR, Günther A, Majeed RW (2019). Provenance for biomedical ontologies with RDF and Git. Stud Health Technol Inform.

[18] Stöhr MR, Günther A, Majeed RW (2019). Verifying data integration configurations for semantical correctness and completeness. Stud Health Technol Inform.

[19] Kadioglu D, Breil B, Knell C, Lablans M, Mate S, Schlue D, Serve H, Storf H, Ückert F, Wagner T, Weingardt P, Prokosch H (2018). Samply.MDR - A metadata repository and its application in various research networks. Stud Health Technol Inform.

[20] Dugas M, Neuhaus P, Meidt A, Doods J, Storck M, Bruland P, Varghese J (2016). Portal of medical data models: information infrastructure for medical research and healthcare. Database (Oxford).

[21] Stöhr MR, Majeed RW, Günther A (2018). Using RDF and Git to realize a collaborative metadata repository. Stud Health Technol Inform.

[22] Miller E (2005). An introduction to the resource description framework. Bul Am Soc Inf Sci Tech.

[23] Pastor-Sanchez J, Martínez-Mendez F, Rodríguez-Muñoz J (2009). Advantages of thesaurus representation using the Simple Knowledge Organization System (SKOS) compared with proposed alternatives. Inf Res.

[24] Weibel SL, Koch T (2000). The Dublin Core Metadata Initiative. D-Lib Magazine.

[25] (2019). ISO/TS 21526 Health informatics - Metadata repository requirements (MetaRep). International Organization for Standardization.

[26] Halilaj L, Grangel-Gonzalez I, Coskun G, Auer S (2016). Git4Voc: Git-based versioning for collaborative vocabulary development. Proceedings of the 2016 IEEE Tenth International Conference on Semantic Computing (ICSC).

[27] Arndt N, Radtke N, Martin M (2016). Distributed collaboration on RDF datasets using Git. Proceedings of the 12th International Conference on Semantic Systems.

[28] SPARQL Protocol And RDF Query Language (SPARQL). W3C Semantic Web.

[29] Stöhr MR, Majeed RW, Günther A (2018). Metadata import from RDF to i2b2. Stud Health Technol Inform.

[30] Yen P, Bakken S (2009). A comparison of usability evaluation methods: heuristic evaluation versus end-user think-aloud protocol - an example from a web-based communication tool for nurse scheduling. AMIA Annu Symp Proc.

[31] Reen GK, Muirhead L, Langdon DW (2019). Usability of health information websites designed for adolescents: systematic review, neurodevelopmental model, and design brief. J Med Internet Res.

[32] (2020). ISO 9241-110 Ergonomics of human-system interaction - Part 110: interaction principles. International Organization for Standardization.

[33] Tullis T, Stetson J (2004). A comparison of questionnaires for assessing website usability. Proceedings of the Usability Professionals' Association Conference.

[34] Brooke J (1996). SUS: a 'Quick and Dirty' usability scale. Usability Evaluation In Industry.

[35] McLellan S, Muddimer A, Peres S (2012). The effect of experience on System Usability Scale ratings. J Usability Stud.

[36] Bastien JC (2010). Usability testing: a review of some methodological and technical aspects of the method. Int J Med Inform.

[37] GoToMeeting. LogMeIn.

[38] Virzi RA (2016). Refining the Test Phase of Usability Evaluation: How Many Subjects Is Enough?. Hum Factors.

[39] Bangor A, Kortum P, Miller J (2009). Determining what individual SUS scores mean: adding an adjective rating scale. J Usability Stud.

[40] Collaborative Metadata Repository (CoMetaR) Code Repository. GitHub.

[41] Collaborative Metadata Repository (CoMetaR) Web Application. Stöhr MR.

